# Changes in Angiogenesis and Bone Turnover Markers in Patients with Gaucher Disease Developing Osteonecrosis

**DOI:** 10.3390/metabo14110601

**Published:** 2024-11-07

**Authors:** Simona D’Amore, Kenneth Eric Poole, Uma Ramaswami, Derralynn Hughes, Kathleen Page, Antonio Giovanni Solimando, Angelo Vacca, Timothy Martin Cox, Patrick Deegan

**Affiliations:** 1 Department of Medicine, University of Cambridge, Cambridge CB2 0QQ, UK; kenneth.poole@nhs.net (K.E.P.); tmc12@medschl.cam.ac.uk (T.M.C.); patrick.deegan1@nhs.net (P.D.); 2 Department of Precision and Regenerative Medicine—Ionian Pole, School of Medicine, “Aldo Moro” University of Bari, 70124 Bari, Italy; antoniogiovannisolimando@gmail.com (A.G.S.); angelo.vacca@uniba.it (A.V.); 3 Lysosomal Storage Disorders Unit, Royal Free Hospital NHS Foundation Trust, London NW3 2QG, UK; uma.ramaswami@nhs.net (U.R.); derralynnhughes@nhs.net (D.H.)

**Keywords:** Gaucher disease, osteonecrosis, biomarker, osteopontin, matrix metalloproteinases, vascular endothelial growth factor

## Abstract

**Background/Objectives**: Patients with Gaucher disease have a high risk of bone disease, with osteonecrosis representing the most debilitating complication. The pathogenesis of osteonecrosis has not been fully elucidated yet, and there is an unmet need for predictive biomarkers of bone complications. We aimed to assess the utility of angiogenesis and bone turnover biomarkers as predictors of osteonecrosis in Gaucher disease. **Methods**: Angiogenesis and bone turnover biomarkers were measured in 146 Gaucher disease patients (70M:76F, median age 49.5 [IQR 36.7 to 61]) with/without osteonecrosis enrolled in the UK-based registry GAUCHERITE [enrolment 2015–2017]. Receiver-operating characteristic curve analysis was used to compare the osteonecrosis predictive value of angiogenesis and bone turnover biomarkers and determine the optimal cut-off values for each biomarker. **Results**: Sixty-two patients had osteonecrosis before study enrolment, 11 had osteonecrosis during follow-up, and 73 remained osteonecrosis-free. Patients with osteonecrosis showed increased osteopontin and matrix metalloproteinase (MMP)-2 levels and decreased MMP-9 and vascular endothelial growth factor (VEGF)-C compared with those free from osteonecrosis. MMP-9 predicted future osteonecrosis with higher sensitivity and specificity (area under the receiver operating characteristic curve [AUC] 0.84 [95% CI 0.84–0.99]; sensitivity/specificity 82%/75%; cutoff value ≤ 72,420 pg/mL) than osteopontin, MMP-2 and VEGF-C when taken alone. The combination of MMP-9 and VEGF-C further increased the discriminating accuracy. **Conclusions**: The osteopontin–MMPs–VEGF axis is dysregulated in Gaucher disease patients with osteonecrosis. The combination of MMP-9 and VEGF-C circulating levels may serve to identify Gaucher disease patients at risk of osteonecrosis.

## 1. Introduction

**Figure 1:**
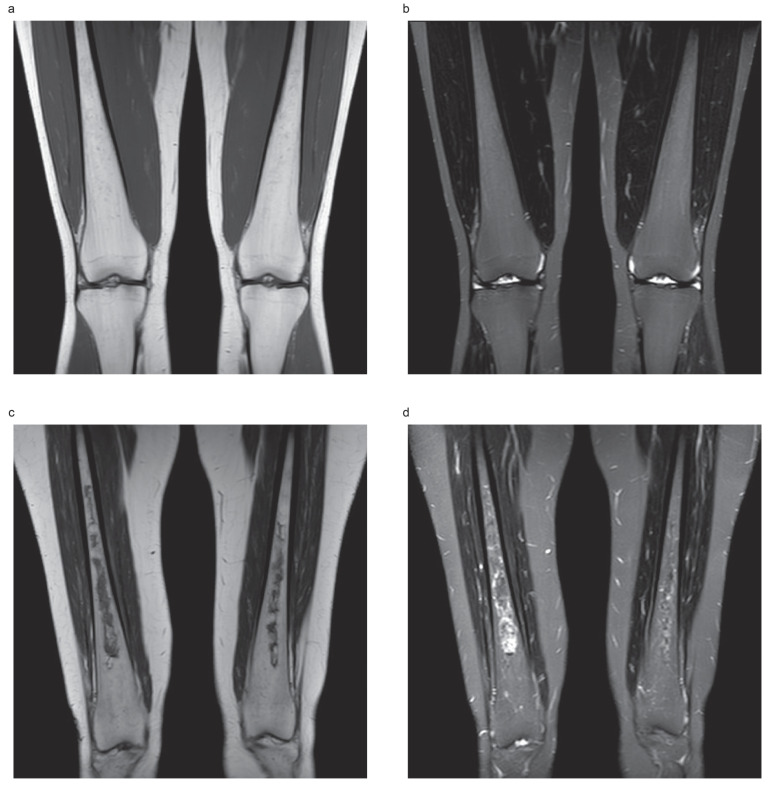
Typical appearance of heterogenous Gaucher marrow fat signals with no evidence of osteonecrosis. (**a**) Coronal T1-weighted image shows several punctate low-signal foci scattered throughout the diaphyseal regions of both femora, corresponding to infiltration of the marrow by Gaucher cells in a 35-year-old woman with Gaucher disease type 1. (**b**) The corresponding coronal Short Tau Inversion Recovery (STIR) image. Typical appearance of an established osteonecrosis. (**c**) Coronal T1-weighted image shows irregularly bordered areas of hypointensity within the diaphyseal regions of both femora, corresponding to fibrosed and sclerosed bone marrow of established bone infarcts in a 50-year-old woman with Gaucher disease type 1. (**d**) The same areas show serpiginous inner rim of hyperintensity on the corresponding coronal Short Tau Inversion Recovery (STIR).

Gaucher disease is an inborn error of metabolism caused by biallelic mutations in the glucocerebrosidase gene (GBA1), leading to deficiency of the enzyme glucocerebrosidase and the progressive accumulation of its substrate (glucocerebroside, Gb1) and its downstream metabolite (glucosylsphingosine, lyso-Gb1) in the lysosomes of mononuclear phagocytes. This results in systemic infiltration by Gaucher cells (glycolipid-laden macrophages) and chronic inflammation [1]. A systemic phenotype arises involving multiple organs (liver, spleen, bone marrow and, occasionally, the lungs): this condition is known as type 1 (non-neuronopathic) Gaucher disease; in neuronopathic fulminant (type 2) and chronic (type 3) Gaucher disease, progressive neurodegenerative manifestations occur [1]. Although the visceral changes can be dramatic in untreated patients, the most debilitating symptoms result from a complex pattern of bone involvement that is usually poorly responsive to enzyme replacement treatment (ERT) and commonly involves osteonecrosis (also known as avascular necrosis) at long bones (tibia, humerus, femur) and vertebrae [2,3,4]. Figure 1 shows examples of heterogenous Gaucher bone marrow fat signals (no osteonecrosis) and established bone osteonecrosis.

The mechanisms underlying the pathogenesis of osteonecrosis remain poorly understood and are believed to involve multiple mechanisms [5,6]. An association of osteonecrosis risk with splenectomy, a palliative measure used before the introduction of enzyme treatment, has been suggested—although the nature of this association remains controversial [2,5,7]—as well as with other risk factors such as anaemia [8], GBA1 genotype (compound heterozygous N370S/other genotype), history of osteonecrosis prior to treatment initiation, type of ERT, and high residual lyso-Gb1 levels [9].

Mounting evidence suggests that the bioactive lipids produced by Gaucher cells also play an important role in the modulation of the function of other cell types, including bone marrow cells and endothelial cells [10,11,12]. Alteration in the blood capillary endothelium has been described in the skin biopsies of patients with Gaucher disease types 2 and 3 [10]. In the bone marrow of patients with Gaucher disease type 1, changes in the vasculature (such as increased microvascular density and abnormal microvessel architecture and function) correlate with the abnormal expression of angiogenetic factors [increased levels of angiopoietins (ANGPTs) and low expression of vascular endothelial growth factors (VEGFs)] [11]. Finally, in a murine model of neuronopathic Gaucher disease, it was suggested that the reduction of cerebral vascularisation was associated with defective angiogenesis due to impaired endothelial cytokinesis and migration related to the accumulation of lyso-Gb1 [12]. Taken together, this evidence suggests that endothelial cells’ dysfunction may play a role in several complications of Gaucher disease, such as osteonecrosis and pulmonary vascular disease.

Understanding the interplay between angiogenesis and the pathogenesis of osteonecrosis could reveal novel pathogenic mechanisms and targetable pathways relevant not only to Gaucher disease but also to other conditions associated with the development of osteonecrosis, such as haemoglobinopathies (e.g., sickle cell disease), corticosteroid use and connective tissue disorders (e.g., systemic lupus erythematosus) [13], thus potentially leading to an unifying model for the pathophysiology of osteonecrosis.

The Gaucher Investigative Therapy Evaluation (GAUCHERITE) is a UK clinical cohort of 251 (250 at the time of the study) Gaucher disease patients with longitudinal observation and collated imaging studies built to enable in-depth clinical phenotyping of the disease, including bone manifestations [14]. In the present study, we aimed to elucidate the interplay of angiogenic and bone turnover molecules in the pathogenesis of osteonecrosis in Gaucher disease and their value as predictive biomarkers of osteonecrosis. To this end, we measured circulating angiogenic and bone turnover factors in Gaucher disease patients free from osteonecrosis and in those who experienced osteonecrosis either before study entry or during the observation phase [14].

## 2. Materials and Methods

### 2.1. Patients

Of the 250 Gaucher patients enrolled in the GAUCHERITE cohort (Clinicaltrials.gov: NCT03240653) at the time of this study, 232 had serum samples collected between May 2015 and January 2018 that were available for analysis. Of this subset, 16 patients were <18 years old at the time of recruitment and excluded from the study to avoid a confounding effect due to age-specific variability in bone and angiogenesis biomarkers (i.e., periods of growth and development with high rates of angiogenesis) [15,16,17,18]. The clinical data and imaging of the remaining 216 Gaucher patients were reviewed to stratify patients according to their osteonecrosis status (presence/absence of osteonecrosis and presence/absence of osteonecrosis during the study). Gaucher disease patients without osteonecrosis had no history of previous symptomatic events nor evidence of osteonecrosis through imaging. A further 4 patients with incident osteonecrosis were excluded from this study since they did not have samples contemporaneous to the event (they either had only samples before the event [n = 2] or before and after the event [n = 1]) or had other complications such as osteomyelitis (n = 1).

**Figure 2:**
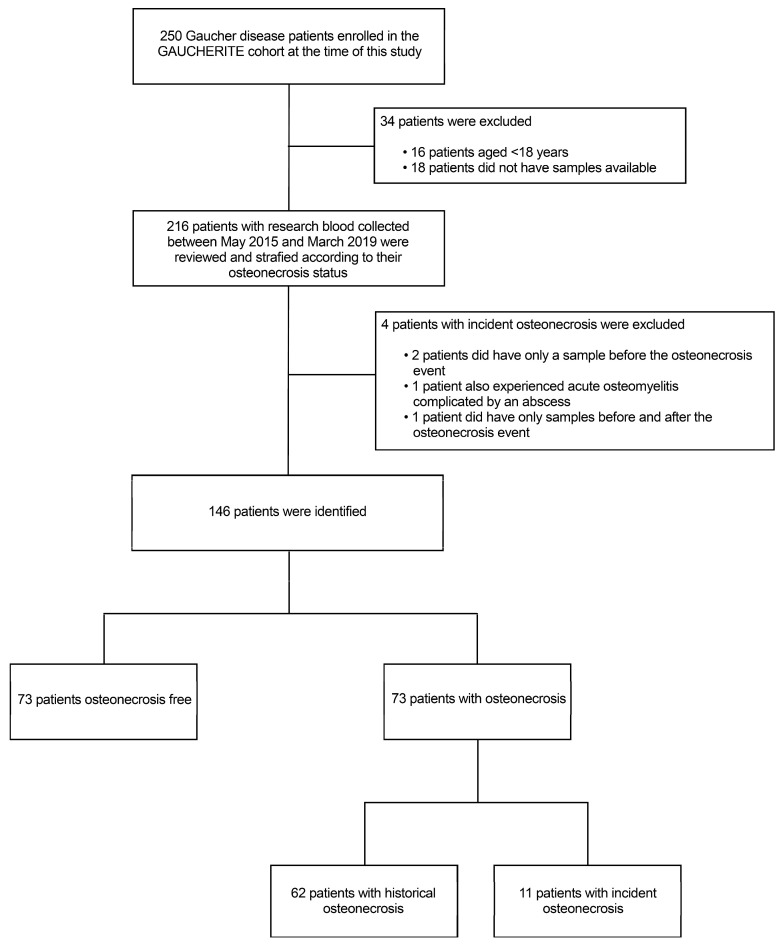
Strobe flow chart shows patient selection. Of the 250 patients assessed for eligibility, 16 were excluded from this study because of their age (<18 years) and 18 because they did not have samples available for the analysis. Two hundred and sixteen patients were reviewed and stratified according to their osteonecrosis status. Of these, 150 age- and sex-matched patients were identified: 62 patients had a history of osteonecrosis and 15 patients had incident osteonecrosis during follow-up, while 73 patients remained free from osteonecrosis. Four patients with incident osteonecrosis were excluded from the final analysis since they either had only samples collected before or after the osteonecrosis event. Another patient also had osteomyelitis and was therefore excluded.

The final study cohort consisted of 73 osteonecrosis cases—classified into 62 patients with a prior history of osteonecrosis and 11 patients who had incident osteonecrosis during follow-up—and 73 patients free from osteonecrosis (randomly selected). Before performing the analyses, we checked that the cohort was age- and sex-matched (as age and sex imbalances would have constituted a confounding factor for the analyses). For patients who had incident osteonecrosis, blood samples at different time points were available for analysis as follows: 6 ± 2 months before osteonecrosis (n = 8), at the time of osteonecrosis as documented by the MRI scan (n = 11) and 6 ± 2 months after the event (n = 8) (see Figure 2 for details).

GBA1 genotyping was performed as part of the clinical phenotyping in the entire cohort by sequencing the genomic DNA at the University of Manchester Centre for Integrated Genomic Medical Research as previously described [14].

The baseline characteristics of patients with no osteonecrosis, prior history of osteonecrosis and incident osteonecrosis during follow-up are shown in Table 1.

### 2.2. Blood Sampling and Serum Storage

Written informed consent from patients was obtained during their routine visits. Following consent, additional research blood samples were collected and centrifuged for 7 min at 4000 rpm (3200 RCF). After link-anonymisation, refrigerated serum samples were shipped and stored at a controlled temperature at the Department of Clinical Biochemistry (Cambridge University Hospitals NHS Foundation Trust, Cambridge, UK), an accredited laboratory for diagnostics and monitoring sampling. Freezing and thawing cycles were minimised by preparing aliquots.

Research blood was collected from patients at the same time as routine blood and urine and transported, stored, accessed and processed in accordance with national legislation relating to the use and storage of human tissue for research purposes, as set out in the 2004 Human Tissue Act [19] and the 2006 Human Tissue (Scotland) Act [20].

### 2.3. Biochemistry

Full blood count, bone metabolism, 25-hydroxyvitamin D, fibrinogen and biomarkers of Gaucher disease activity [21] [ferritin, enzyme chitotriosidase, CC chemokine pulmonary and activation-regulated chemokine (PARC/CCL18), angiotensin-converting enzyme (ACE)] contemporaneous to research blood were also measured.

### 2.4. Biomarker Assays

The Meso Scale Discovery assays (MSD, 1601 Research Blvd., Rockville, MD 20850-3173, USA) used for this study were all sandwich electrochemiluminescence immunoassays. MSD provided a plate pre-coated with up to ten capture antibodies on independent and well-defined spots. Sample, standards or quality controls (QCs) were added to the plate and incubated at room temperature with mixing. After washing, a cocktail of detection antibodies was added to the plate, which was then incubated at room temperature with mixing. After washing, MSD read buffer was added to the plate. This provided the appropriate chemical environment for electrochemiluminescence. The plate was loaded into the MSD Sector S 600 instrument (MSD, 1601 Research Blvd., Rockville, MD 20850-3173, USA) for analysis. Inside the MSD instrument, a voltage was applied to the plate electrodes, which caused the labels bound to the electrode surface to emit light. The instrument measured the intensity of emitted light from each well-defined spot to afford a quantitative measure of each of the cytokines present in the sample. Results were calculated using MSD Discovery Workbench software 4.0. All reagents and standards were supplied by MSD.

Standards, QCs and samples were loaded onto the plates using a Tecan Evo100 liquid-handling robot (Tecan Group Ltd., Seestrasse 103, 8708 Männedorf, Switzerland). Washing of the plates was performed using a Thermo Scientific WellWash Versa plate washer (Thermo Fisher Scientific, 168 Third Avenue, Waltham, MA 02451, USA). Three specific QC samples were used for this study. These were prepared in bulk at the start of the study and stored as single-use aliquots frozen at below −70 °C and thawed on the day of use. QC 1 and QC 2 were dilutions of the MSD-supplied standard material. QC 3 was a pool of anonymised serum samples. All three controls were analysed at the beginning (after the standards) and end (after the samples) of each plate.

Assay Specifics: MSD Bone Panel 2 (product number: K15147C-2; assays: osteocalcin, osteonectin, osteopontin; sample dilution: 1:20; sample volume: 25 μL; incubation time for samples: 2 h; incubation time for detector antibody: 1 h); MSD Human MMP 3-plex Ultra-Sensitive (product number: K15034C-2; assays: MMP-1, MMP-3, MMP-9; sample dilution: 1:10; sample volume: 25 μL; incubation time for samples: 2 h; incubation time for detector antibody: 2 h); MSD Human TIMP-1 (product number: K151JFC-2; assays: TIMP-1; sample dilution: 1:100; sample volume: 25 μL; incubation time for samples: 2 h; incubation time for detector antibody: 2 h); MSD V-plex Human Angiogenesis Panel 1 (product number: K150190D; assays: VEGF-A, VEGF-C, VEGF-D, Tie-2, Flt-1, PIGF, β-FGF; sample dilution: 1:2; sample volume: 25 μL; incubation time for samples: 2 h; incubation time for detector antibody: 2 h); MSD Human MMP 2-Plex Ultra-Sensitive (product number: K15033C-2; assays: MMP-2, MMP-10; sample dilution: 1:2; sample volume: 12.5 μL; incubation time for samples: 2 h; incubation time for detector antibody: 2 h); MSD R-Plex Human OPG (product number: F21ZK-3; assays: osteoprotegerin; sample dilution: 1:2; sample volume: 12.5 μL; incubation time for samples: 1 h; incubation time for detector antibody: 1 h).

Biomarker analyses were performed in 2019 in bulk to avoid batch effects.

### 2.5. Bone Disease Assessment

The presence and approximate onset of osteonecrosis were based on prior clinical history or current evidence of characteristic symptoms and confirmed by supportive radiological evidence of osteonecrosis on MRI or plain radiographs [2,14]. In patients with asymptomatic osteonecrosis (i.e., radiological evidence of active bone ischaemia in the absence of characteristic symptoms), the imaging date was considered as the approximate date of onset. The presence of fragility fracture was based on a positive history of fracture “that occurred as a result of a minimal trauma, such as a fall from standing height or less, or no identifiable trauma”, as defined by the World Health Organization [22]; in addition, radiologic imaging on thoracic and lumbar radiographs and spine MRI were also reviewed to detect subclinical or undiagnosed vertebral fractures [23]. The presence and grading of osteoarthritis were performed according to the Kellgren–Lawrence scale, where osteoarthritis is diagnosed with a KL grade of two or greater [24] for both hip and knee radiographs taken as non-weight-bearing standard views and interpreted in their anteroposterior view. The presence of Erlenmeyer flask deformity was defined by a ratio of the width of diametaphysis 4 cm from the physeal plate divided by the physeal plate width ≥ 0.58 [25].

### 2.6. Gaucher Disease Severity Scoring System

The Gaucher disease type 1 disease severity scoring system (GD-DS3) is a validated disease scoring system for adults with Gaucher disease type 1 and is based on bone, hematologic and visceral domains. GD-DS3 expresses the disease burden and response to treatment and is defined as follows: severe (GD-DS3 > 9); marked (GD-DS3 6–9); moderate (GD-DS3 3–6); mild (GD-DS3 < 3.00) [26].

### 2.7. Statistical Analysis

Cases (patients with historical and incident osteonecrosis) and controls (patients free from osteonecrosis) were matched with respect to sex and age. Comparisons between groups (osteonecrosis-free; historical osteonecrosis; incident osteonecrosis) were assessed by Kruskal–Wallis one-way analysis of variance followed by Dunn’s post-hoc test and Pearson’s chi-squared test. Correlation coefficients were used to determine the relationship between the variables. Receiver-operating characteristic (ROC) curve analysis estimated the sensitivity/specificity and optimal cut-off values (highest sensitivity/specificity) of biomarkers in predicting osteonecrosis risk in patients with incident osteonecrosis during follow-up. All statistical analyses were conducted using NCSS software (v21.0.2; NCSS, LCC); the null hypothesis was rejected when the *p*-value was ≤0.05.

## 3. Results

### 3.1. Characteristics of the Subjects

One-hundred and forty-six patients with Gaucher disease (70M:76F, median age 49.5 [IQR 36.7 to 61]) were included in the analysis (Table 1). Of these, 133 had a clinical diagnosis of non-neuronopathic type 1 Gaucher disease and 13 of neuronopathic type 3 Gaucher disease. N370S/other GBA1 genotype was the commonest genotype among the study cohort (65/146 [45%]). Other GBA1 variants included N370S/L444P (27/146 [19%]), N370S/N370S (23/146 [16%]), L444P/L444P (6/146 [6%]), L444P/other (5/146 [3%]) and other genotypes either in homozygosity (1/146 [<1%]) or heterozygosity (16/146 [11%]). Sixty-two (42.5%) patients had sustained one or multiple osteonecrosis before enrolment, 11 (7.5%) patients developed a new osteonecrosis during follow-up, and 73 (50%) patients remained free from osteonecrosis. Overall, 174 osteonecrosis events—some involving multiple skeletal sites—were observed in the 73 patients with historical/incident osteonecrosis included in this study. The most common site of osteonecrosis was the femur (68/73 patients [93%] had sustained at least one femoral osteonecrosis).

Patients with either prior-history or incident osteonecrosis were much more likely to have a younger age at Gaucher disease presentation (median age 8.2 years [IQR 4.2 to 20.3] and 6.5 years [IQR 4.4 to 25.3], respectively) than those without osteonecrosis (median age 26.5 years [IQR 13.8 to 37.8], *p*-value < 0.001 (Table 1)), confirming that early onset is associated with a more severe disease course [5].

Patients with either prior-history or incident osteonecrosis showed a significantly higher incidence of fragility fractures (16/62 [26%] and 4/11 [36%], respectively), osteoarthritis (35/62 [56.5%] and 10/11 [91%]), orthopaedic procedures (28/62 [45%] and 7/11 [64%], respectively) and presence of Erlenmeyer flask deformity (40/62 [64.5%] and 7 [64%]) compared with those free from osteonecrosis (fragility fractures 4/73 [5.5%], *p* = 0.001; osteoarthritis 31/73 [42%], *p* = 0.007; orthopaedic procedures 7/73 [10%], *p* < 0.001; Erlenmeyer flask deformity 24/73 [33%], *p* = 0.001) while the presence of lytic lesions was similar across groups (Table 1).

Among Gaucher disease type 1 patients, GD-DS3 scores were calculated near the study entry to assess disease severity (i.e., bone, hematologic and visceral domains and overall disease severity score). Median GD-DS3 scores significantly differed (*p* < 0.001) by osteonecrosis status and were within the mild severity range among patients without osteonecrosis (median score 2 [IQR 1–4]), while they were in the moderate severity range among patients with prior-history and incident osteonecrosis (median score 4 [IQR 3 to 6] and 6 [IQR 4 to 9], respectively).

Two type 3 Gaucher disease patients in the historical osteonecrosis group had undergone haematopoietic stem cell transplantation. Splenectomy was reported in 25 of the 62 (40%) patients with a prior history of osteonecrosis and in 5 of the 11 (45%) patients with incident osteonecrosis, while only 7 of the 73 (10%) patients with no osteonecrosis had prior history of splenectomy (*p* < 0.001). Most splenectomies had occurred prior to 1991, in the pre-ERT era.

While the three groups differed very little regarding the age at which treatment was initiated, patients with a prior history of osteonecrosis and incident osteonecrosis had a longer interval between Gaucher disease presentation and treatment initiation with Gaucher-specific treatment (median 15.4 years [IQR 4.4–24] and 16.9 [IQR 1–30.4], respectively) than those free from osteonecrosis (median 3 years [IQR 0.9–10.3], *p* < 0.001). However, the time of treatment initiation has changed since ERT was approved in 1991. It must be noted that 6 of the 11 (55%) patients with incident osteonecrosis and 39 of the 62 (63%) with prior history of osteonecrosis were diagnosed before 1991, compared with 22 of the 73 (29%) patients free from osteonecrosis (*p*-value = 0.001). Additionally, there was an excess of bone manifestations in the osteonecrosis groups with respect to treatment initiation: 38 of the 62 patients with a prior history of osteonecrosis (61%) and 7 of the 11 with incident osteonecrosis (64%) had a history of a major bone event (i.e., osteonecrosis, fragility fracture, lytic lesion or orthopaedic procedure) prior to starting treatment, compared with 3 of 73 of those free from osteonecrosis (4%, *p*-value < 0.001).

Plasma biochemistry was similar across groups, except for a significantly increased platelet count and angiotensin-converting enzyme levels in patients with a prior history of osteonecrosis (median 194 [IQR 160–252]; median 51 [IQR 38–80], respectively) than those free from osteonecrosis (median 178 109/L [IQR 145–219], *p* = 0.043; median 40 109/L [IQR 25–58], *p* = 0.040; details in Table 2). As expected, platelet counts were higher in splenectomised patients (median 286 109/L [IQR 236 to 330]) compared with those not splenectomised (median 173 109/L [IQR 146 to 209], *p* < 0.001).

### 3.2. Circulating Markers of Angiogenesis and Bone Turnover Markers in Gaucher Disease Patients Stratified by Osteonecrosis Status

**Figure 3:**
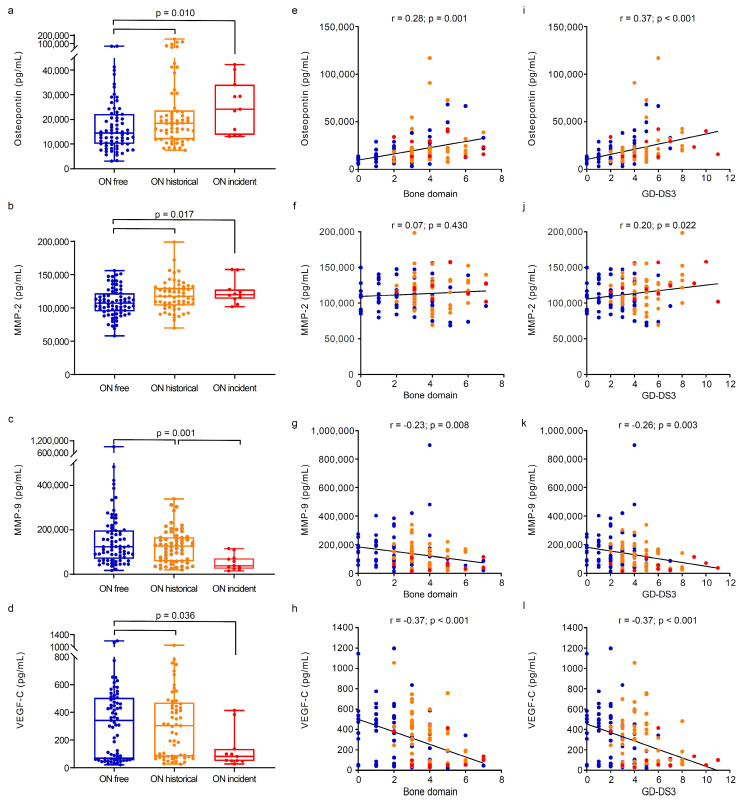
Differences in bone and angiogenesis biomarkers in Gaucher disease patients according to their osteonecrosis status. Analysis of differences between groups indicated that patients with Gaucher disease who experienced either historical or incidental osteonecrosis had greater levels of (**a**) osteopontin (Kruskal–Wallis one-way ANOVA on ranks test: *p*-value = 0.010) and (**b**) MMP-2 (Kruskal–Wallis one-way ANOVA on ranks test: *p*-value = 0.017) and lower levels of (**d**) VEGF-C (Kruskal–Wallis one-way ANOVA on ranks test: *p*-value = 0.036) compared with those osteonecrosis-free. Patients with incident osteonecrosis had lower levels of (**c**) MMP-9 (Kruskal–Wallis one-way ANOVA on ranks test: *p*-value = 0.001) compared with those free from osteonecrosis and those with historical events. Boxes include the data between first and third quartiles, the central bar indicates the median, and the whiskers show minimum and maximum values. The dots represent all patients. In patients with Gaucher disease type 1, osteopontin was positively correlated to (**e**) bone disease (r = 0.28, *p*-value = 0.001) and (**i**) disease severity (r = 0.37, *p*-value < 0.001), while MMP-2 was positively correlated to (**j**) disease severity (r = 0.20, *p*-value = 0.022) but not with (**f**) bone disease. MMP-9 and VEGF-C were negatively correlated to (**g**,**h**) bone disease (r = −0.23, *p*-value = 0.008 and r = −0.37, *p*-value < 0.001, respectively) and (**k**,**l**) disease severity (r = −0.26, *p*-value = 0.003 and r = −0.37, *p* < 0.001). The correlation between continuous variables was assessed with Spearman’s rank correlation coefficient. GD-DS3 = Gaucher disease type 1 disease severity scoring system; MMP = matrix metalloproteinase; ON = osteonecrosis; VEGF-C = vascular endothelial growth factor C. (●) Osteonecrosis-free; (●) historical osteonecrosis; (●) incident osteonecrosis.

We then studied the circulating markers of angiogenesis and bone turnover in Gaucher disease patients stratified by osteonecrosis status (Figure 3 and Table 3). For patients with incident osteonecrosis, samples contemporaneous to the approximate onset of osteonecrosis were used for this analysis.

Patients with both historical and incident osteonecrosis showed a significant increase in the levels of osteopontin (median 18,474 pg/mL [IQR 11,951.25 to 23,575.25] and median 24,113 pg/mL [IQR 13,754 to 34,091], respectively) and MMP-2 (median 116,699.5 pg/mL [IQR 102,860.8 to 129,362.3] and median 119,970 pg/mL [IQR 113,784 to 127,694], respectively) and a decrease in VEGF-C (median 304.3 pg/mL [IQR 81.05 to 471.4] and median 80.9 pg/mL [IQR 49.1 to 136.9], respectively) compared with subjects free from osteonecrosis (respectively, median 14,462 [IQR 10,057 to 22,202.5], *p* = 0.010, and median 342.4 pg/mL [IQR 64.6 to 505.4]), *p* = 0.036, (Figure 3a,b,d). Decreased levels of MMP-9 were also observed in patients with incident osteonecrosis (median 38,711 pg/mL [IQR 24,822 to 72,420]) compared with patients free from osteonecrosis (median 121,223 pg/mL [IQR 68,135.5 to 195,583.5]) and those with historical osteonecrosis (median 126,312 pg/mL [IQR 58,997–165,988.3], *p* = 0.001, Figure 3c).

**Figure 4:**
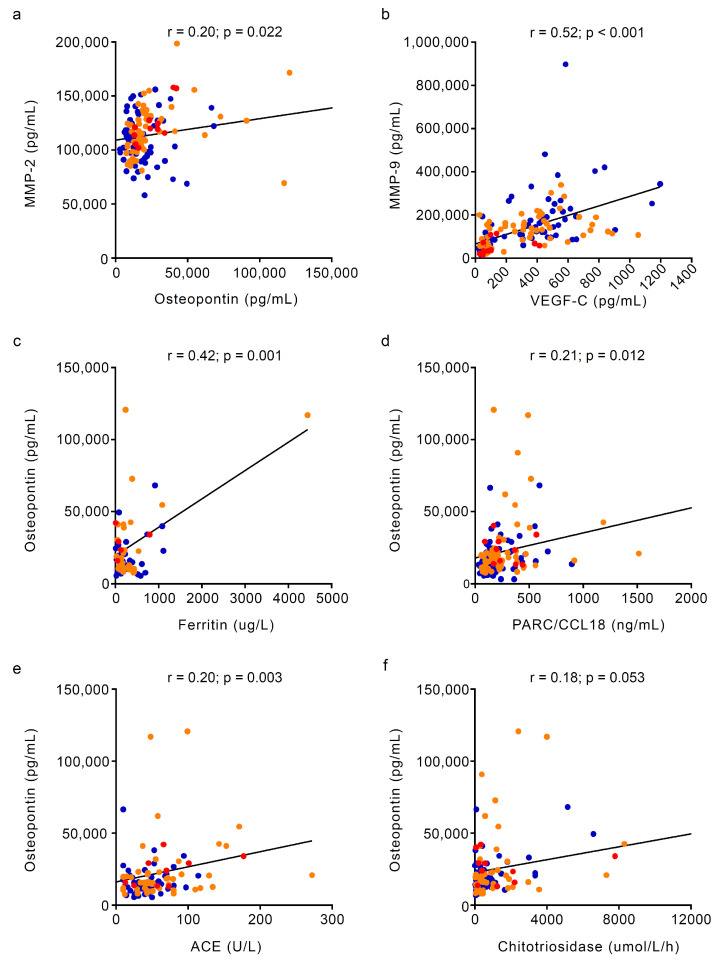
Correlations of bone and angiogenesis biomarkers in Gaucher disease patients. In the overall population, a positive correlation was observed between (**a**) MMP-2 and osteopontin (r = 0.20, *p*-value = 0.022) and (**b**) MMP-9 and VEGF-C (r = 0.52, *p*-value < 0.001). Osteopontin was also positively correlated with other markers of Gaucher disease severity, such as (**c**) ferritin (r = 0.42, *p*-value = 0.001), (**d**) PARC/CCL18 (r = 0.21, *p*-value = 0.012), (**e**) ACE (r = 0.20, *p*-value = 0.044) and (**f**) chitotriosidase (r = 0.18, *p* = 0.053). The correlation between continuous variables was assessed with Spearman’s rank correlation coefficient. (●) Osteonecrosis-free; (●) historical osteonecrosis; (●) incident osteonecrosis. ACE = angiotensin-converting enzyme; MMP = matrix metalloproteinase; ON = osteonecrosis; PARC/CCL18 = CC chemokine pulmonary and activation-regulated chemokine; VEGF-C = vascular endothelial growth factor C.

A positive correlation was found between MMP-2 and osteopontin (r = 0.20, *p*-value = 0.022) and between MMP-9 and VEGF-C (r = 0.52, *p*-value < 0.001, Figure 4a,b).

In patients with Gaucher disease type 1, osteopontin positively correlated to bone disease domain (r = 0.28, *p*-value = 0.001) and GD-DS3 (r = 0.37, *p*-value < 0.001), while MMP-2 was positively correlated to GD-DS3 (r = 0.20, *p*-value = 0.022) but not to bone disease. MMP-9 and VEGF-C were negatively correlated to both bone disease (r = −0.23, *p*-value = 0.008 and r = −0.37, *p*-value < 0.001, respectively) and GD-DS3 (r = −0.26, *p*-value = 0.003; r = −0.37, *p* < 0.001; details in Figure 3).

In the overall population, only osteopontin showed a positive correlation with other markers of disease severity, such as ferritin (r = 0.42, *p*-value = 0.001), PARC/CCL18 (r = 0.21, *p*-value = 0.012), ACE (r = 0.20, *p*-value = 0.044) and chitotriosidase (r = 0.18, *p* = 0.053, Figure 4). The lack of strong correlations between angiogenesis and bone markers with established Gaucher disease-specific biomarkers of disease activity suggests a specific effect of osteonecrosis on their circulating levels rather than a disease severity effect.

### 3.3. Sensitivity and Specificity of Angiogenesis and Bone Biomarkers for Detecting Osteonecrosis

**Figure 5:**
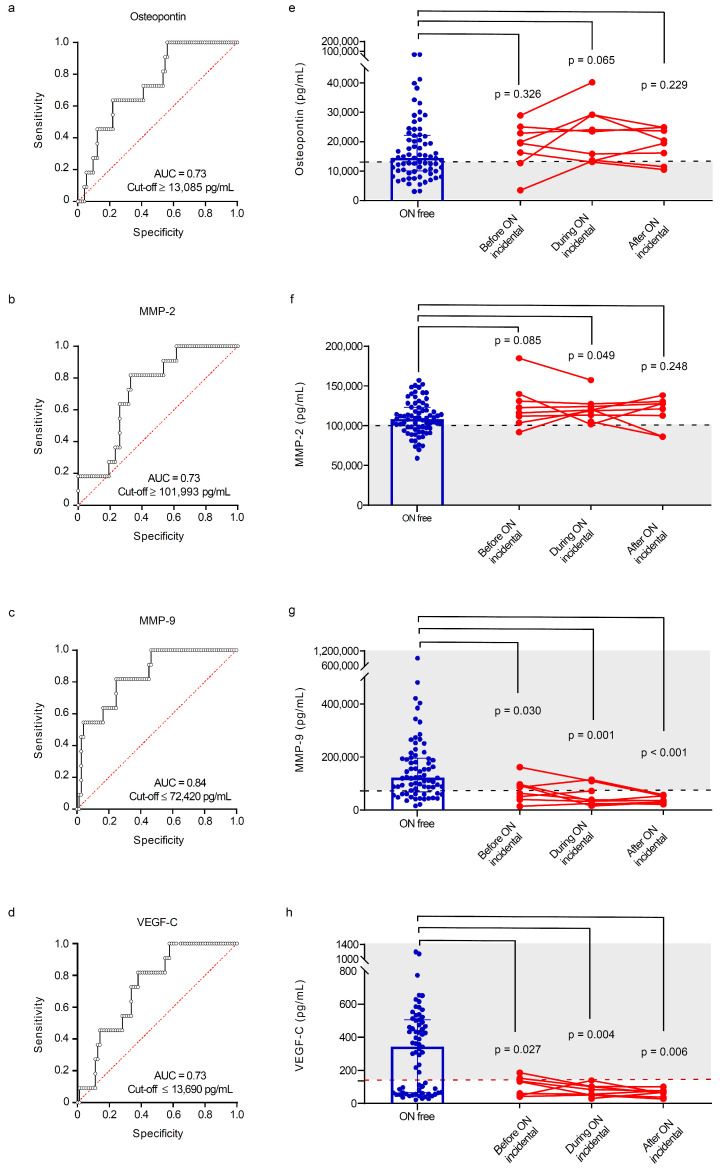
Accuracy of bone and angiogenesis biomarkers in predicting osteonecrosis in Gaucher disease patients. ROC curve analysis with area under the curve, sensitivity and specificity of (**a**) osteopontin, (**b**) MMP-2, (**c**) MMP-9 and (**d**) VEGF-C in the prediction of osteonecrosis. Cut-offs for (**e**) osteopontin, (**f**) MMP-2, (**g**) MMP-9 and (**h**) VEGF-C in patients who remained free from osteonecrosis and those who sustained osteonecrosis during follow-up. For each marker, individual values are shown before, during and after the event. AUC = area under the curve; MMP = matrix metalloproteinase; ON = osteonecrosis; VEGF-C = vascular endothelial growth factor C. (●) Osteonecrosis-free; (●) incident osteonecrosis.

ROC curve analyses were performed to estimate optimal cut-off values, sensitivity and specificity for angiogenesis and bone markers to predict the risk of osteonecrosis in Gaucher disease patients with incident osteonecrosis. We found similar area under the receiver operating characteristic curves (AUCs) for osteopontin (0.73 [95% CI 0.56–0.85]; sensitivity/specificity 100%/44%; cut-off value ≥ 13,085 pg/mL), MMP-2 (0.73 [95% CI 0.57–0.84]; sensitivity/specificity 100%/38%; cut-off value ≥ 101,993 pg/mL) and VEGF-C (0.73 [95% CI 0.57–0.84]; sensitivity/specificity 82%/62%; cut-off value ≤ 13,690 pg/mL; details in Figure 5a,b,d). MMP-9 showed the highest accuracy in the prediction of osteonecrosis events (AUC of 0.84 [95% CI 0.68–0.92]; sensitivity/specificity 82%/75%; cutoff value ≤ 72,420 pg/mL; details in Figure 5c).

We further explored the predictive value of osteopontin, MMP-2, MMP-9 and VEGF-C by testing their abundance in a subgroup of patients with incident osteonecrosis where blood samples were available before, during and after the event and comparing their values with those of patients who remained free from osteonecrosis. Notably, MMP-9 and VEGF-C were significantly lower in patients with incident osteonecrosis before (*p* = 0.030 and *p* = 0.027, respectively), during (*p* = 001 and *p* = 0.004, respectively) and after (*p* < 0.001 and *p* = 0.006, respectively) the event (Figure 5g,h), suggesting their potential as predictive biomarkers of ON in subjects at risk. Moreover, using the combination of MMP-9 and VEGF-C and the cut-offs suggested by the ROC curve analysis, seven out of the eight patients (88%) with samples before and during osteonecrosis were already showing values of MMP-9 and/or VEGF-C below the cut-offs: the combination of these two biomarkers could effectively predict the onset of osteonecrosis; however, these results need to be validated in a larger cohort of patients with multiple data points and long-term follow-up.

## 4. Discussion

Nontraumatic osteonecrosis is a debilitating and progressive complication of Gaucher disease that continues to represent a challenge to physicians since it is frequently associated with the risk of joint collapse and no disease-modifying treatment exists [3]. This reflects the lack of understanding of its underlying pathophysiology, which is a prerequisite to developing novel therapeutic options for this disabling skeletal manifestation. However, while the exact pathogenesis of osteonecrosis has not yet been elucidated and remains controversial, most theories point toward the interaction of multiple factors, such as vascular dysfunction, alterations of the bone–cell physiology and their microenvironment, risk factors (e.g., corticosteroid use, alcohol abuse), and genetic predisposition, leading to increased occurrence of ischaemia, bone death and, subsequently, the initiation of the bone healing process [27]. In Gaucher disease, the progressive accumulation of glycolipids within macrophages results in the release of pro-inflammatory chemokines and cytokines that might also affect the balance of bone resorption and formation, thus contributing to the pathophysiology of osteonecrosis [28].

Different molecules, including growth factors and MMPs, have emerged as key actors of bone remodelling and osteonecrosis under pathological conditions: these secreted factors physiologically maintain the homeostasis between bone matrix degradation and reconstruction, ensuring the maintenance of the composition and mechanical characteristics of bone tissue during bone repair [29].

In this study, we investigated the changes in circulating markers of bone turnover and angiogenesis occurring during and after osteonecrosis in a cohort of patients with Gaucher disease stratified according to their osteonecrosis status.

Osteopontin levels were found to be increased in both patients with historical and incident osteonecrosis compared with those free from osteonecrosis. Osteopontin, also known as early T-lymphocyte activation 1 protein (ETA1), is a member of the SIBLING (Small Integrin-Binding Ligand, N-linked Glycoprotein) proteins that are secreted by several cell types, such as bone cells (i.e., osteoblasts, osteoclasts and osteocytes) and inflammatory cells (e.g., macrophages); osteopontin is a substrate of MMPs (MMP-9 and, possibly, MMP-2), and tartrate-resistant acid phosphatase (TRACP), which is increased in Gaucher disease [30,31]. Osteopontin promotes the migration and adhesion of bone marrow mesenchymal cells, osteoblasts and osteoclasts on the bone resorption surface, where it regulates bone development and maintenance [32]. Osteopontin is also a proinflammatory mediator that activates inflammatory cells and osteoclasts and inhibits mineralisation [29,31,33]. Osteopontin levels thus correlate with a higher bone turnover and lower bone mineral density and are associated with the risk of several bone-related diseases, such as osteoarthritis and osteoporosis [32]. The elevated levels of osteopontin observed in patients with both historical and incident osteonecrosis, as well as its correlations with several markers of disease activity, suggest that bone remodelling goes beyond the acute event, potentially having a role in the morphological formation and reconstruction of bone tissue after the necrotic damage.

MMPs levels were differently expressed in patients with osteonecrosis (elevated MMP-2 and decreased MMP-9) when compared with those free from osteonecrosis. MMP-2 and -9 are primarily produced by the osteoblasts and osteoclasts, respectively, and play a complex role in bone formation and resorption, thus being implicated in the maintenance of bone integrity and quality [29,34]. MMP-2 and MMP-9 also stimulate angiogenesis, a key component of wound healing and bone repair [35,36,37]. MMP-9-deficient mice showed severe defects in skeletal development and diminished angiogenesis, thus leading to reduced restoration of microvascular perfusion capacity in response to ischemia [38]; moreover, MMP-9 mRNA levels were lower in a mouse model of glucocorticoid-induced osteonecrosis compared with controls [39], while its upregulation (e.g., after treatment with exosomes released by bone mesenchymal stem cells in steroid-induced femoral head necrosis) promoted osteogenesis and improved osteonecrosis [40]. Last, high levels of MMP-9 along with an imbalance in the MMP-9/TIMP-1 ratio have been found in patients with idiopathic, alcohol- and steroid-induced nontraumatic osteonecrosis of the femoral head and correlated with the development and severity of osteonecrosis [41]. Elevated levels of MMP-2 and decreased levels of MMP-9 have also been observed in patients with mucopolysaccharidosis (MPS), suggesting that altered MMP expression may contribute to joint/bone abnormalities in other lysosomal storage disorders [42,43]. Therefore, our results suggest that Gaucher disease patients with osteonecrosis might suffer from an imbalance in mechanisms governing extracellular matrix catabolism, tissue remodelling, and angiogenesis: these processes are needed to ensure adequate bone regeneration, although the underlying mechanism may differ depending on the disease/model.

We also found that Gaucher patients with osteonecrosis had a reduction in the circulating levels of VEGF-C (a critical mediator of angiogenesis) compared with subjects who remained free from osteonecrosis. VEGFs have emerged as prominent modulators of angiogenic–osteogenic coupling (required to obtain efficient vascularisation and bone formation during bone repair): physiological VEGF levels modulate angiogenesis and maintain bone homeostasis by modulating osteoblast differentiation and bone resorption [44,45,46,47,48]. Moreover, several genetic polymorphisms of VEGF are associated with susceptibility to nontraumatic osteonecrosis [49,50,51], while a reduction in circulating VEGF-C is associated with the development of osteonecrosis of the jaw in patients receiving bisphosphonate [52].

Intriguingly, MMP-9 and VEGF-C were strongly correlated, and their changes were evident before, during and after the occurrence of osteonecrosis, thus suggesting that the molecular events that lead to their suppression (and to osteonecrosis) occur before the acute event: these circulating factors can therefore act as predictive biomarkers of osteonecrosis.

Taken together, our results offer a novel set of potential biomarkers of osteonecrosis in Gaucher disease as well as novel insights into the underlying mechanisms involved in osteonecrosis, which may represent new targets for treatment.

This study has several limitations. First, the relatively small sample size, primarily due to the rarity of Gaucher disease, may have resulted in underpowered tests for those variables that returned non-statistically significant results. Second, this study only evaluated the value of angiogenetic and bone turnover biomarkers in Gaucher disease, which may limit the generalisability of the results. Third, the retrospective nature of this study limited the ability to evaluate trends in biomarker profiles beyond the post-acute setting after incident osteonecrosis. Last, research samples were stored for different lengths of time (from 1 to 4 years); therefore, we cannot exclude an effect of different storage times on the analyses.

## 5. Conclusions

In conclusion, the understanding of the mechanisms that underlie the onset of osteonecrosis in GD patients is in its infancy. A timely diagnosis of osteonecrosis, as well as the capability to identify patients at risk, is a priority to prevent this painful GD complication and its progression. Hence, there is a need for biomarkers to stratify patients at a higher risk of osteonecrosis. To the best of our knowledge, this is the first evidence that an increase in circulating osteopontin and MMP-2 and a reduction in MMP-9 and VEGF-C levels are directly linked to osteonecrosis in Gaucher disease. Our data point to several potential candidate biomarkers of atraumatic osteonecrosis, with combined MMP-9 and VEGF-C being the most promising. These results prompt the need for validation in a larger longitudinal cohort.

## Figures and Tables

**Table 1:** Demographic and clinical characteristics of the study cohort by osteonecrosis status.

Variable	Osteonecrosis—Free	Osteonecrosis—Historical	Osteonecrosis—Incident	*p*-Value
N	73 (36M:37F)	62 (28M:34F)	11 (6M:5F)	
Age, years, median (IQR)	47 (34.4–60.3)	47.7 (35.8–60.9)	49.5 (39.2–66.8)	0.446
GENOTYPE, N %				
Homozygous N370S/N370S	18 (25%)	4 (6.5%)	1 (9%)	0.036 +
Homozygous L444P/L444P	3 (4%)	6 (10%)	-	
Homozygous other/other ^a^	1 (1%)	-	-	
Heterozygous N370S/L444P	14 (19%)	11 (18%)	2 (18%)	
Heterozygous N370S/other ^a^	29 (39.7%)	32 (52%)	4 (36.5%)	
Heterozygous L444P/other ^a^	1 (1%)	4 (6.5%)	-	
Heterozygous other/other ^a^	7 (10%)	5 (8%)	4 (36.5%)	
TYPE OF GAUCHER DISEASE, N %				
Type 1	68 (93%)	55 (89%)	10 (91%)	0.665 +
Type 3	5 (7%)	7 (11%)	1 (9%)	
Age at Gaucher disease presentation, years, median (IQR)	26.5 (13.8–37.8)	8.2 (4.2–20.3)	6.5 (4.4–25.3)	<0.001 1, 2 ≠ 0 ‡
OSTEONECROSIS, N %				
Symptomatic osteonecrosis events	-	36	9	0.001 +
Asymptomatic osteonecrosis events	-	30	11	
Age at first osteonecrosis, years, median (IQR)	-	25.07 (11.88–37.43)	21.95 (12.6–37.8)	0.845 #
FRACTURE, N %				
No	69 (94.5%)	46 (74%)	7 (64%)	0.001 +
Yes	4 (5.5%)	16 (26%)	4 (36%)	
Age at first fracture, years, median (IQR)	57.65 (16.85–61.59)	45.39 (25.15–52.95)	23.9 (13.7–34.8)	0.176
OSTEOARTHRITIS, N %				
No	42 (58%)	27 (43.5%)	1 (9%)	0.007 +
Yes	31 (42%)	35 (56.5%)	10 (91%)	
Age at osteoarthritis diagnosis, years, median (IQR)	43 (34.3–53.7)	41.8 (33.7–52.4)	40.4 (32.9–53.9)	0.885
ORTHOPEDIC PROCEDURE, N %				
No	66 (90%)	34 (55%)	4 (36%)	<0.001 +
Yes	7 (10%)	28 (45%)	7 (64%)	
Age at first orthopaedic procedure, years, median (IQR)	46.1 (13–55.2)	33.3 (14.83–47.65)	46.9 (19.1–54.3)	0.281
**Variable**	**Osteonecrosis—Free**	**Osteonecrosis—Historical**	**Osteonecrosis—Incident**	***p*** **-Value**
ERLENMEYER FLASK DEFORMITY (EFD), N %				
No	49 (67%)	22 (35.5%)	4 (36%)	0.001 +
Yes	24 (33%)	40 (64.5%)	7 (64%)	
Age at EFD diagnosis, years, median (IQR)	37.2 (25.5–48)	36.02 (22.8–49.35)	45.5 (34.3–49.4)	0.567
LYTIC LESION, N %				
No	71 (97%)	58 (94%)	10 (91%)	0.474 +
Yes	2 (3%)	4 (6%)	1 (9%)	
Age at first lytic lesion, years, median (IQR)	48.0 (27.3–68.8)	30.6 (9.0–42.4)	47.2 (47.2–47.2)	0.343
GAUCHER DISEASE TYPE 1 SEVERITY SCORING SYSTEM (GD-DS3)				
Bone domain, median (IQR)	2 (0.3–3)	4 (3–5)	4.5 (3–6.3)	<0.001 1, 2 ≠ 0
Haematologic domain, median (IQR)	0 (0–0.75)	0 (0–0)	0 (0–1)	0.435
Visceral domain, median (IQR)	0 (0–1)	0 (0–2)	1 (0–3.3)	0.057
Total Gaucher DS3 score, median (IQR)	2 (1–4)	4 (3–6)	6 (4–9)	<0.001 1, 2 ≠ 0 ‡
BONE MARROW TRANSPLANT, N %				
No	73 (100%)	60 (97%)	11 (100%)	0.253 +
Yes	-	2 (3%)	-	
Age at bone marrow transplant, years, median (IQR)	-	6.3 (1.7–10.8)	-	-
SPLENECTOMY, N %				
No	66 (90%)	37 (60%)	6 (55%)	<0.001 +
Yes	7 (10%)	25 (40%)	5 (45%)	
Age at splenectomy, years, median (IQR)	27 (24.09–54.05)	11.72 (5.02–20.76)	15.4 (7.2–21.7)	0.011 1 ≠ 0 ‡
GAUCHER SPECIFIC TREATMENT, N %				
No	7 (10%)	2 (3%)	-	0.209 +
Yes	66 (90%)	60 (97%)	11 (100%)	
Age at Gaucher specific treatment initiation, years, median (IQR)	34.5 (25.4–45.01)	29.25 (20.15–42.4)	35.4 (20.4–45.9)	0.555
Time from Gaucher disease presentation and treatment initiation, years, median (IQR)	3 (0.9–10.3)	15.4 (4.4–24.3)	16.9 (1–30.4)	<0.001 1, 2 ≠ 0 ‡
BONE TREATMENT, N %				
No	28 (38%)	20 (32%)	3 (27%)	0.652 +
Yes	45 (62%)	42 (68%)	8 (73%)	
**Variable**	**Osteonecrosis—** **Free**	**Osteonecrosis—Historical**	**Osteonecrosis—Incident**	***p*-Value**
STEROID TREATMENT, N %				
No	59 (81%)	52 (84%)	11 (100%)	0.277 +
Yes	14 (19%)	10 (16%)	-	

Continuous variables presented as median (interquartile range, IQR). Categorical variables presented as numbers (%). Kruskal–Wallis one-way ANOVA test; ‡ Dunn’s post-hoc test (0 = osteonecrosis-free; 1 = historical osteonecrosis; 2 = incident osteonecrosis); + Chi-square test; # Mann–Whitney test. ^a^ Other is defined as any allele other than N370S or L444P.

**Table 2:** Biochemistry parameters of the study cohort by osteonecrosis status.

Variable	Osteonecrosis—Free	Osteonecrosis—Historical	Osteonecrosis—Incident	*p*-Value
Haemoglobin, g/L, median (IQR)	139 (129–146.5)	136 (131–150)	137 (128–152)	0.873
White cell count, ×10^9^/L, median (IQR)	5.7 (4.7–7.5)	6.4 (5.0–8.4)	6.6 (3.7–9)	0.319
Platelets, ×10^9^/L, median (IQR)	178 (145–219)	194 (160–252)	237 (152–325)	0.043 2 ≠ 0 ‡
Alkaline phosphatase, U/L, median (IQR)	67.5 (55.8–84)	67 (56–83)	73 (55.5–95.3)	0.864
Calcium, mmol/L, median (IQR)	2.3 (2.3–2.4)	2.4 (2.3–2.4)	2.3 (2.3–2.4)	0.229
Corrected calcium, mmol/L, median (IQR)	2.4 (2.3–2.5)	2.4 (2.3–2.5)	2.4 (2.4–2.4)	0.300
Vitamin D, ug/L, median (IQR)	61.1 (43–84.5)	58.3 (39.8–82.0)	56.8 (40.1–67.7)	0.749
Vitamin B12, ng/L, median (IQR)	405 (310–556)	399 (317.5–507)	425 (356–538)	0.861
Ferritin, ug/L, median (IQR)	153 (76.3–535.3)	190 (119.2–371.1)	73.8 (27.8–460.5)	0.332
ACE, U/L, median (IQR)	40 (25–58)	51 (38–80)	66 (35–87)	0.040 2 ≠ 0 ‡
Chitotriosidase, umol/L/h, median (IQR)	303 (125.5–878)	519 (254.5–1301.5)	479.5 (199.5–2138.3)	0.128
PARC/CCL18, ng/mL, median (IQR)	149.5 (104.3–251.5)	209 (117.3–378.8)	224 (171–391.8)	0.055
Fibrinogen, g/L, median (IQR)	2.67 (2.02–3.17)	2.59 (2.16–2.83)	2.92 (2.66–3.1)	0.141

Continuous variables presented as median (interquartile range, IQR). ACE = angiotensin-converting enzyme; PARC/CCL18 = CC chemokine pulmonary and activation-regulated chemokine. Kruskal–Wallis one-way ANOVA test; ‡ Dunn’s post-hoc test (0 = osteonecrosis-free; 1 = historical osteonecrosis; 2 = incident osteonecrosis).

**Table 3:** Bone and angiogenetic biomarkers of the study cohort by osteonecrosis.

Variable	Osteonecrosis—Free	Osteonecrosis—Historical	Osteonecrosis—Incident	*p*-Value
Osteonectin, pg/mL, median (IQR)	769,231 (508,562.5–971,934)	785,403.5 (585,508–1,003,565)	322,027 (124,400.5–857,722.5)	0.306
Osteopontin, pg/mL, median (IQR)	14,462 (10,057–22,202.5)	18,474 (11,951.25–23,575.25)	24,113 (13,754–34,091)	0.010 1, 2 ≠ 0 ‡
Osteocalcin, pg/mL, median (IQR)	28,629 (19,786–34,474.5)	26,751 (19,038–35,658.5)	28,341 (17,304–45,209)	0.576
MMP-1, pg/mL, median (IQR)	4945 (1699–11,427)	5378.5 (2889.75–8872)	3058 (980–6332)	0.253
MMP-2, pg/mL, median (IQR)	10,7193 (95,016.5–122,201)	116,699.5 (102,860.8–129,362.3)	119,970 (113,784–127,694)	0.017 1, 2 ≠ 0 ‡
MMP-3, pg/mL, median (IQR)	12,663 (7838.5–18,749.5)	12,721.5 (9052.5–17,771.25)	12,314 (10,295–18,204)	0.627
MMP-9, pg/mL, median (IQR)	121,223 (68,135.5–195,583.5)	126,312 (58,997–165,988.3)	38,711 (24,822–72,420)	0.001 2 ≠ 1,0 ‡
MMP-10, pg/mL, median (IQR)	1113 (745.5–1408)	1095 (892.75–1425.25)	919 (571–974)	0.058
TIMP-1, pg/mL, median (IQR)	299,558 (215,000.5–34,8074)	306,410.5 (190,432.5–360,033.5)	190,183 (161,362–285,583)	0.147
Osteoprotegerin, pg/mL, median (IQR)	297 (229–381)	314 (251.75–407)	284 (239–406)	0.589
FGF, pg/mL, median (IQR)	3 (1.85–5.1)	3.8 (2.4–6.125)	3 (2–3.7)	0.156
FLT-1, pg/mL, median (IQR)	92.7 (70.75–113.55)	92.65 (72.1–116.8)	83.6 (67.1–127.8)	0.834
PLGF, pg/mL, median (IQR)	6.8 (5.5–8.2)	6.1 (4.875–7.5)	5.8 (5.1–7.3)	0.098
TIE-2, pg/mL, median (IQR)	5162 (4314.5–5693.5)	4786.5 (3964.75–5752.25)	4820 (4348–5088)	0.634
VEGF-A, pg/mL, median (IQR)	185.7 (84.55–298.25)	182.35 (69.225–504.68)	73.1 (37–127)	0.056
VEGF-C, pg/mL, median (IQR)	342.4 (64.6–505.4)	304.3 (81.05–471.4)	80.9 (49.1–136.9)	0.036 2 ≠ 1, 0 ‡
VEGF-D, pg/mL, median (IQR)	1246 (1001.5–1880.5)	1260 (966–1735)	854 (696–1318)	0.072

Continuous variables presented as median (interquartile range, IQR). FGF = fibroblast growth factor; FLT-1 = vascular endothelial growth factor receptor 1; MMP = matrix metalloproteinase; PLGF = placental growth factor; TIE-2 = angiopoietin-1 receptor; TIMP-1 = tissue inhibitor of metalloproteinase 1; VEGF = vascular endothelial growth factor. Kruskal–Wallis one-way ANOVA test; ‡ Dunn’s post-hoc test (0 = osteonecrosis-free; 1 = historical osteonecrosis; 2 = incident osteonecrosis).

## Data Availability

The data that support the findings of this study are held by the University of Cambridge under a Medical Research Council-approved Data Management Policy. Restrictions apply and data are not publicly available: applications from bona fide organisations will be considered by the University of Cambridge and clinical governance of Cambridge University NHS Foundation Trust Hospitals and subject to approval by the GAUCHERITE Consortium Management Committee.
